# High-density expression profiling of renal cell carcinomas from Saudi Arabia: a preliminary study

**DOI:** 10.1186/1471-2164-15-S2-P36

**Published:** 2014-04-02

**Authors:** Sajjad Karim, Hasan MA Farsi, Hans-Juergen Schulten, Jaudah A Al-Maghrabi, Nuha A Alansari, Alaa A Albogmi, Mamdooh A Gari, Adeel GA Chaudhary, Adel M Abuzenadah, Mohammed H Al Qahtani

**Affiliations:** 1 Center of Excellence in Genomic Medicine Research, King Abdulaziz University, PO BOX 80216, Jeddah 21589, Saudi Arabia; 2 Department of Urology, Faculty of Medicine, King Abdulaziz University, Jeddah, Saudi Arabia; 3 Department of Pathology, Faculty of Medicine, King Abdulaziz University, Jeddah, Saudi Arabia; 4 Department of Pathology, King Faisal Specialist Hospital and Research Center, Jeddah, Saudi Arabia

## Background

Renal cell carcinoma (RCC) is the most common malignancy of the adult kidney, comprising 3-4% of all human cancers, ranked sixth-leading cause of cancer death and incidences are increasing worldwide [1]. If detected in early stages, it is potentially curable by surgical resection; however, only a fraction of metastatic RCC is responsive to treatment. The molecular events leading to disease onset and progression are not well understood and needs investigations.

## Materials and methods

We performed whole gene expression profiling of RCC (n=4) and normal renal tissue (n=5) using Affymetrix HuGene 1.0 ST arrays. We retrieved selected expression data from NCBI’s “Gene Expression Omnibus” database (GSE781, GSE7023, and GSE6344) for comparative analysis [2]. Ingenuity Pathway Analysis (Ingenuity System), a genome-wide biological pathway analysis package, was used to find significantly molecular networks and pathways associated with kidney cancer.

## Results

We identified 1515 differentially expressed significant genes, 967 up and 548 down regulated, with cutoff false discovery rate ≤ 0.05 and a fold change > 2; comparing RCC with normal kidney tissues. The most significantly upregulated genes were topoisomerase DNA II binding protein 1 (TOPBP1), tryptophan 2,3-dioxygenase (TDO2), forkhead box M1 (FOXM1), ankyrin repeat domain 13A (ANKRD13A), and potassium inwardly-rectifying channel JI (KCNJ1) whereas downregulated genes were nephrosis2 (NPHS2), uromodulin (UMOD), calbindin1 (CALB1), solute carrier family12 (SLC12A3), plasminogen (PLG). We also found 781 genes to be common, comparing our data with retrieved data. IPA based canonical pathway analysis shown Atherosclerosis signaling, LXR/RXR activation, GM-CSF signaling, Notch Signaling, Leukocyte Extravasation Signaling pathway to be significantly associated with our kidney cancer cases and this finding is in accordance with other finding [3-5].

## Conclusions

Present study provides an initial overview of differentially expressed genes in kidney cancer of Saudi Arabian patients using whole transcript, high-density expression arrays. Comparative analysis suggest that even though data set is small but has a potential source for novel biomarker for kidney cancer and may offer unique biological insights into these tumors. In conclusion, it is important to study gene expression profiles comprehensively to extract more sophisticated biological interpretations.

*Authors would like to acknowledge the KACST*, *Riyadh*, *Saudi Arabia* (*Project ID: 10BIO1258-03 and 10BIO1073-03*) *for funding the research.*
